# The protracted waste crisis and physical health of workers in Beirut: a comparative cross-sectional study

**DOI:** 10.1186/s12940-017-0240-6

**Published:** 2017-04-11

**Authors:** Rami Z. Morsi, Rawan Safa, Serge F. Baroud, Cherine N. Fawaz, Jad I. Farha, Fadi El-Jardali, Monique Chaaya

**Affiliations:** 1 Faculty of Medicine, American University of Beirut, Beirut, Lebanon; 2 Department of Health Management and Policy, Faculty of Health Sciences, American University of Beirut, Beirut, Lebanon; 3 Department of Epidemiology and Population Health, Faculty of Health Sciences, American University of Beirut, Riad El-Solh, Beirut 1107 2020, PO Box 11-0236, Beirut, Lebanon

**Keywords:** Waste, Waste management, Dumpsites, Waste burning, Physical health, Workers

## Abstract

**Background:**

Since July 2015, Lebanon has been experiencing a waste management crisis. Dumpsites in inhabited areas and waste burning have emerged due to the waste accumulation, further adding to the gravity of the situation. However, the association between the crisis and health of the population has not been scientifically reported.

**Methods:**

A comparative cross-sectional study was conducted to assess whether exposure to open dumpsites and waste burning is associated with acute health symptoms. The study sample included 221 male workers between the ages of 18–60 years selected from two areas chosen based on their proximity to a garbage dumpsite and waste burning. 110 workers were exposed to a garbage dumpsite and waste burning, and 111 workers were not. Data were collected via a face-to-face interview using a newly developed validated structured questionnaire. Chi-square tests were used to check for statistically significant differences between exposure and covariates. Multivariable analyses using multiple logistic regression were used to compare health symptoms between exposed and unexposed workers adjusting for potential confounders.

**Results:**

The prevalence of acute health symptoms was greater among the exposed workers than the non-exposed workers, including gastrointestinal, respiratory, dermatological and constitutional symptoms. Controlling for confounding variables, such as age, insurance, family support, residence near dumpsite, work site, and smoking, a minimum odds ratio (OR) of 4.30 was obtained when comparing the exposed population to those non-exposed.

**Conclusion:**

The strong association between improper waste management and physical health calls for immediate attention by the government, stakeholders and community members to find optimal solutions for this waste management crisis and set immediate priority interventions such as regular waste collection, volume reduction and recycling performance improvement. However, the long recall period may have underestimated our results.

## Background

From July 17, 2015 till end of September, heaps of garbage crowded the residential areas and streets in Lebanon. Civic protests broke out throughout the country calling for a clean-up of the streets and asking for a permanent solution to the waste management dilemma the country has been facing for years [1]. Temporary solutions were made, eventually resulting in the reemergence of the crisis in August 2016. Due to the lack of a proper national recycling plan, waste management remains as one of the most challenging development-related issues that the government is facing. Historically, municipalities have managed disposal and refuse collection. As a consequence of civil unrest, municipalities have been unable to provide a much needed service and until recently slow burning and uncontrolled dumping on hillsides and seashores, particularly in industrial areas such as Dawra and Dekwaneh, have become common practices for solid waste disposal in Lebanon resulting in serious land, sea and air pollution problems [2].

The change in weather conditions and prolonged duration of the crisis reached a critical stage, resulting in multiple claims regarding health implications and infectious illnesses, including accumulation and leakage of toxins into water sources and spreading of pathogens including, but not limited to, *Vibrio cholerae* and hepatitis [3]. While leakage into water sources can be compensated by a high-quality water system, this leakage, however, could be concerning given the inefficient water system in Lebanon. Municipal water supplied to households in Lebanon was reported to be turbid, possibly because of infrequent monitoring of the municipal water quality [4].

Improper disposal of garbage, specifically burning wastes in the open, poses serious danger on health. Some of the most dangerous pollutants emitted are dioxins, particle pollution, carbon monoxide and volatile organic compounds. Dioxins are persistent, bio-accumulative and toxic pollutants that settle on plants, thus building up in the food chain and resulting in harmful effects. Some of the adverse effects of dioxins include suppression of immune systems, disruption on hormonal system and cancer [5–7]. Particle pollutants are microscopic particles that can aggravate respiratory conditions such as asthma and bronchitis and are associated with cardiac arrhythmias and heart attacks. Carbon monoxide can cause neurologic symptoms in people exposed to significant burning of garbage; these symptoms include headache, fatigue, nausea and vomiting. Inhaling volatile organic compounds can cause headaches, loss of coordination, nausea, damage to the liver, kidney and central nervous system and irritation to the eye, nose and throat. Hence, the broad spectrum of adverse effects caused by emission of these compounds into the atmosphere calls for serious restructuring of waste management in Lebanon [8].

Current research indicates that burning wastes can increase the risk of heart disease, aggravate respiratory ailments such as asthma and emphysema and cause rashes, nausea, headaches or diarrhea, damages in the nervous system, kidney or liver, in the reproductive system and development system [9, 10]. For instance, burning of polystyrene polymers, such as foam cups or meat trays, releases styrene gas that is absorbed through the skin and lungs, resulting in harmful effects ranging from a spectrum of acute symptoms to severe life-changing problems [5]. Moreover, the acid and chemicals emitted by trash fires can cause severe bronchoconstriction in asthmatics [11]. Other long-term effects caused by exposure to a landfill include congenital abnormalities and malformations [12].

In Lebanon, waste accumulation on the streets and other sites has led to the emergence of unlicensed landfills and dumpsites in inhabited areas [3]. Improper waste management is a public health concern with a large spectrum of outcomes. However, data concerning the magnitude of the ongoing waste crisis, such as the prevalence of acute health symptoms resulting from improper waste management, is still scarce and has not been scientifically reported. This study aimed to assess whether exposure to dump sites and waste burning is associated with poor physical health, defined by the occurrence of acute health symptoms. Acute symptoms have a rapid onset and a short course. It includes, but is not limited to, general systemic symptoms such as nausea, skin allergies, dyspnea – shortness of breath – and asthma attacks.

## Methods

### Research design

A comparative cross-sectional study of 221 male workers aged 18–60 years was conducted in the northern suburbs of the capital city.

A total of 110 participants was taken from workplaces in Dawra, a region in the Northern suburbs of the capital Beirut, characterized by patriarchal industrial areas, and in proximity (100 m radius) of a major open dump, where the waste burning also occurred. A comparison group of 111 workers was taken from Dekwaneh, another industrial area in the suburbs of Beirut, close to Dawra and where no garbage dump existed within a 100 m radius. Workers who have been working in the chosen industrial areas of similar composition for at least 3 months were interviewed. Foreign workers, mentally handicapped and physically disabled workers were excluded to minimize any communication problems.

A walk-through survey method was adopted to select a random sample of workers in each of the two areas. All consecutive shops (mechanic, electrician, retail of auto spare parts, companies such as BOSCH) in the blocks were selected. Upon consent of the employer to approach his/her employees, the investigators inquired from the employees whose birthdays were closest to the date of interview and selected him. The research team member(s) filled out the structured questionnaire accordingly on behalf of the selected employees.

### Measures

We defined acute symptoms as those that have a rapid onset and a short course; the outcomes encompassed 22 physical symptoms from major systems, including non-specific constitutional symptoms. These systems included the gastrointestinal systems, integumentary system/skin, and upper respiratory system. Other constitutional symptoms such as dizziness, nausea, and headache were noted as well.

Indicators for the acute symptoms included the presence of the symptoms based on a recall period of 2 months prior to the interview, duration, severity, treatment, and absence from work (in days).

Other co-variates included the socioeconomic characteristics of workers (age, educational level, marital status, occupation, income sufficiency, and health insurance status); exposure related variables such as working site (indoors or outdoors), residency area (whether there is a garbage dump present in the vicinity of their residence) and the use of protective measures (eg. mask, etc.); health-related variables including presence of chronic conditions such as diabetes, asthma, cancer, and smoking status.

### Data collection

After gaining consent from the Institutional Review Board (IRB) at the American University of Beirut, data were collected from the participants through a structured questionnaire. The employer was first approached to take permission for interviewing the workers. Upon his approval, the workers were then randomly selected based on the last-birthday sampling method, where the available employees were called upon by the employer and were asked for their birthdays. If the employer refused to give consent, then no replacement of the shop was made. The employees with the birthdays closest to the date of interview at the time being were selected for the study, and the research team member(s) asked for the employees’ verbal consent. Upon consenting, the research team member(s) proceeded with surveying each worker in a more private place in the shop or office, avoiding any third person not directly involved with the face-to-face interview or research team.

### Data analysis

We analyzed the quantitative data using the Statistical Package for Social Sciences (SPSS) v.23. The survey results were summarized and presented as the percentage of participants who had a response to each statement. Frequency distributions and 95% confidence intervals (95% CI) were calculated to estimate relevant prevalence rates of acute symptoms.

All co-variates were cross-tabulated with exposure status and Chi-square tests performed to check for statistically significant differences. The presence of acute symptoms was compared according to exposure status. Unadjusted ORs and their 95% confidence intervals were reported. The symptoms chosen for multivariable analysis were based on the number of outcomes per symptom and their significance. The adjusted ORs for each symptom were then calculated using multiple logistic regression including the confounding variables found to significantly affect the data at the bivariate level. Finally, bivariate analysis was conducted to compare the distribution of acute conditions between the exposed sample and the non-exposed sample. Non-parametric tests such as Mann–Whitney *U* test were then used to confirm whether there was an association between exposure to waste and the distribution of symptoms. A *p*-value of < 0.05 was considered statistically significant.

## Results

Table 1 presents the characteristics of the participants by exposure status. The two groups were similar with respect to age, education, marital status, work shift duration, and income sufficiency. Exposed workers were more likely than non-exposed to be living near a dumpsite (42.7% vs 22.7%), and to work indoors (48.2% vs. 26.1) and to be the main support to their family (81.0% vs. 65.5%).

**Table 1:** Demographic and health-related characteristics for exposed and non-exposed workers

Variable	Non-exposed (%; *n* = 111)	Exposed (%; *n* = 110)	*p*-value^a^
I. Demographic characteristic			
*Age*			0.138
18–33	31.5	40.4	
34–49	28.8	29.4	
50–60	39.6	30.3	
*Residence near a dump site*			0.001
No	77.3	57.3	
Yes	22.7	42.7	
*Education*			0.473
Primary level	25.2	26.4	
Secondary level	52.3	50.0	
University level	22.5	23.6	
*Work hours*			0.368
≤ 8 h	49.5	47.3	
> 8 h	50.5	52.7	
*Workplace*			0.000
Indoors	26.1	48.2	
Outdoors	48.6	24.5	
Indoors and outdoors	25.2	27.3	
*Marital status*			0.425
Married	34.2	33.0	
Not married	65.8	67.0	
*Support to family*			0.005
No	34.5	19.0	
Yes	65.5	81.0	
*Income*			0.416
Insufficient	45.5	44.0	
Sufficient	54.5	56.0	
II. Heath-related characteristic			
*Presence of Chronic Disease*			0.300
No	77.5	80.4	
Yes	22.5	19.6	
*Smoking*			0.009
No	54.1	38.2	
Yes	45.9	61.8	
*Insurance*			0.075
No	19.1	27.3	
Yes	80.9	72.7	

^a^Chi-squared; *p*-value < 0.05 is considered significant

With regard to the health-related demographic characteristics, the majority of both non-exposed and exposed workers did not have a chronic disease, such as diabetes mellitus, hypertension, etc. A significantly higher proportion of exposed workers were smokers (61.8% vs. 45.9%). The majority of both non-exposed and exposed workers had insurance.

### Prevalence of acute physical symptoms with exposure to waste

Multivariable analysis was used to control for the potential confounders that were found to be significant with exposure and outcomes at the bivariate level. These confounders included residence near a dumpsite, place of work (indoors, outdoors, or both), family support and smoking status. Age and insurance were also controlled for, to minimize any effect on their association with the workers’ physical health.

Table 2 shows the results of different logistic regression models for the association between each acute physical symptom and exposure to waste, after adjusting for confounders. Workers exposed to waste burning and dump sites have significantly greater odds of having fatigue (OR = 22.48; 95% CI 9.34–54.09), headache (OR = 16.88; 95% CI 7.85–36.31), and insomnia (OR = 10.64; 95% CI 3.09–36.67) among general constitutive symptoms. Exposed workers also have greater odds of gastrointestinal manifestations, including nausea (OR = 9.72; 95% CI 4.74–19.92), diarrhea (OR = 4.30; 95% CI 1.65–11.18), and vomiting (OR = 4.74; 95% CI 1.53–14.68). Being exposed was also significantly associated with respiratory symptoms, including dyspnea (OR = 14.99; 95% CI 6.60–34.01), sneezing (OR = 24.75; 95% CI 8.41–72.80), dry cough (OR = 9.57; 95% CI 4.03–22.70), sputum (OR = 8.79; 95% CI 3.23–23.94), and rhinorrhea (OR = 6.00; 95% CI 2.48–14.56).

**Table 2:** The unadjusted and adjusted odds ratios (ORs) of physical symptoms significantly associated* with exposure

System category	Physical symptom	Non-exposed (%)	Exposed (%)	Unadjusted OR	95% CI (Unadjusted)	Adjusted OR^a^	95% CI (Adjusted)
General							
	*Fatigue*	6.3	65.5	28.150	3.261; 38.070	22.48	9.34; 54.09
	*Headache*	9.0	64.5	18.387	8.615; 39.244	16.88	7.85; 36.31
	*Insomnia*	2.7	23.6	11.143	11.908; 66.546	10.64	3.09; 36.67
Gastrointestinal							
	*Nausea*	10.8	57.3	11.059	5.446; 22.454	9.72	4.74; 19.92
	*Diarrhea*	5.4	21.8	4.884	1.910; 12.489	4.30	1.65; 11.18
	*Vomiting*	3.6	19.3	6.384	2.113; 19.289	4.74	1.53; 14.68
Respiratory							
	*Dyspnea*	7.2	56.4	16.630	7.383; 37. 461	14.99	6.60; 34.01
	*Sneeze*	3.6	51.8	28.769	9.909; 83.529	24.75	8.41; 72.80
	*Dry cough*	6.3	41.8	10.679	4.546; 25.083	9.57	4.03; 22.70
	*Sputum*	4.5	32.7	10.314	3.865; 27.518	8.79	3.23; 23.94
	*Rhinorrhea*	6.3	32.7	7.228	3.050; 17.127	6.00	2.48; 14.56
	*Chest tightness*	0.9	22.7	32.353	4.297; 243.575	^b^	^b^
	*Nose scratch*	0.9	19.1	25.955	3.424; 196.728	^b^	^b^
Dermatological							
	*Sting*	0.9	30.0	47.143	6.313; 352.068	^b^	^b^

^a^adjusted for: smoking, support, place of residence, working place, insurance, and age

^b^Not considered due to low number of outcomes

*all *p*-values were < 0.0001

Moreover, the median number of symptoms for exposed workers was greater than that of non-exposed workers (1 vs. 4). An independent-samples Mann–Whitney *U* test showed that there was a statistically significant difference between the underlying distributions of symptoms in exposed workers as opposed to non-exposed workers.

### Severity of acute physical symptoms among both exposed and non-exposed workers

Nausea, headache and diarrhea were chosen to estimate the severity of the symptoms among the exposure group, out of all the symptoms found to be significant and with prevalence greater than 10%. Around half of those exposed reporting headaches took medication (45.1%), while around 80% of those not exposed reporting headaches took medication. Those exposed who reported nausea had leaves of absence ranging from zero to 30 days, while those not exposed who reported nausea did not take any leaves of absence. Additionally, around a quarter of exposed workers who reported diarrhea visited doctors; around one-tenth of these individuals took leaves of absence. On the other hand, around five percent of those non-exposed who reported diarrhea visited doctors, and none of these individuals took any leave of absence.

## Discussion

The results from this comparative cross-sectional study showed that exposure to open waste dump is associated with a greater likelihood of acute symptoms. These results are consistent with the claims mentioned on the health implications that the improper waste management could result in, such as an increased risk of asthma and increased rate of hospitalization due to respiratory and gastrointestinal manifestations [3].

To the best of our knowledge, no studies have been conducted on the association between garbage exposure and physical health of workers during the waste crisis that extended from July till end of September 2015. Most of the claims were not based on scientific evidence. Moreover, it is the first study documenting and analyzing the type of symptoms occurring as well as the severity. These findings reinforce the negative implications of improper waste management as suggested by other findings in regards to exposure to carcinogenic air toxins such as dioxins linked to diseases like lymphomas, leukemias, and heart disease [13].

This is a rigorous epidemiological study controlling for many potential confounders. One potential variable that was not taken into account is the installment and usage of ventilation systems. We also minimized recall bias by coinciding data collection with the waste crisis. However, a two-month recall period may have underestimated the prevalence and duration of reported symptoms or may have affected the reported data related to medication. Other strengths included selecting a sample of adequate representation of the working population, and increasing the external validity of the study by randomly selecting the workers. The working population chosen was representative of the working Lebanese population, given the age and gender of the sample selected. The socio-demographic characteristics were generally similar to that of the Lebanese population in regards to age groups, smoking, education, marital status and chronic medical conditions [14].

The confidence in the association between garbage exposure and physical health of workers was affected by a number of limitations. One limitation was that documenting symptoms was self-reported and based on recall and not on any objective medical assessment. In addition, the target population did not include vulnerable age groups such as children and the elderly, but rather only included those workers, who were present at the time of data collection. Moreover, workers who were severely affected by the crisis may have not been present at the time of data collection.

### Policy implications

Therefore, our study establishes an association of large magnitude between garbage exposure and physical health. The burden of garbage exposure on physical health is not the only alarming issue; the garbage exposure may also have financial implications, which pose as an economic burden on the Lebanese population. Workers may have also transmitted the infections to other household members, and this may be considered a social burden. Hence, this study stresses the importance of taking strict measures to alleviate any anticipated consequences of the waste management crisis. Although waste management plans are being currently discussed, they are still not optimal to alleviate the symptoms entirely. Instead, they might be resulting in shifting the symptoms elsewhere.

Short-term measures to be considered include industrial waste minimization by adopting measures such as production process modification, volume reduction and recovery. Each municipality can create well-planned landfills or incineration sites located more than 10 km away from inhabited areas, attempt to prevent trash piles from contaminating water sources, routinely measure contaminated water sources for toxins, implement awareness campaigns on recycling and health hazards, and collaborate with the government and non-governmental organizations (NGOs) to achieve better outcomes [3]. It is important to set immediate priority interventions such as regular waste collection, volume reduction and improvement in recycling performance. On an individual level, citizens should also be more aware of protective measures such as recycling, minimizing individual acts of waste burning, and wearing masks, in order to minimize the transmission of pathogens.

Long-term measures include establishing a proper color-coding system for waste segregation, collection, transportation, storage and providing proper training for waste handlers; import and export regulatory regimes should also be established on a policy level as forms of interventions; a regulatory framework and national guidelines should be established under which regional and national treatment/disposal polices are to be developed; an example of a framework first developed by a Dutch non-governmental organization is the Integrated Sustainable Waste Management (ISWM). This waste management plan would optimally include a cohesive tie in the environmental sustainability, economic costs and social acceptance of all potential solutions for the current waste crisis in Beirut [15, 16]. Another multifaceted measure would be the initiation of a national committee in Lebanon for the monitoring and surveillance of proper disposal and garbage treatment practices. The government can also participate by appointing local municipalities with waste management responsibilities, and provide these municipalities with technical and financial support [3]. Lebanon recently finalized the municipalities that potentially need to systematically address this waste management problem in an evidence-based manner. However, our results are timely and critical for these new leaders to urgently take on a more active role. Despite the fact that there might not be an optimal solution for waste disposal, efforts should be directed towards involving all stakeholders in formulating local models and building on the strengths of the city [17].

## Conclusion

It has been established that improper waste management can have an impact on the physical health in the short run. However, if this waste crisis is not contained and managed immediately, it might have other long-term health implications. Therefore, it is important to take necessary measures and implement policies to maintain the situation, to avoid emergence of epidemics and attend to these health consequences before they occur.

## Abbreviations

CI: Confidence interval
ISWM: Integrated Sustainable Waste Management
OR: Odds ratio
